# Supplementary material for: Modulation of Viral Programmed Ribosomal Frameshifting and Stop Codon Readthrough by the Host Restriction Factor Shiftless
Source: Viruses. 2021 Jun 25;13(7):1230. doi: 10.3390/v13071230 (PMC8310280; doi:10.3390/v13071230)
Supplement: Supplementary file 1 [file viruses-13-01230-s001.zip › viruses-1250065-SI.pdf]

Supplementary Material

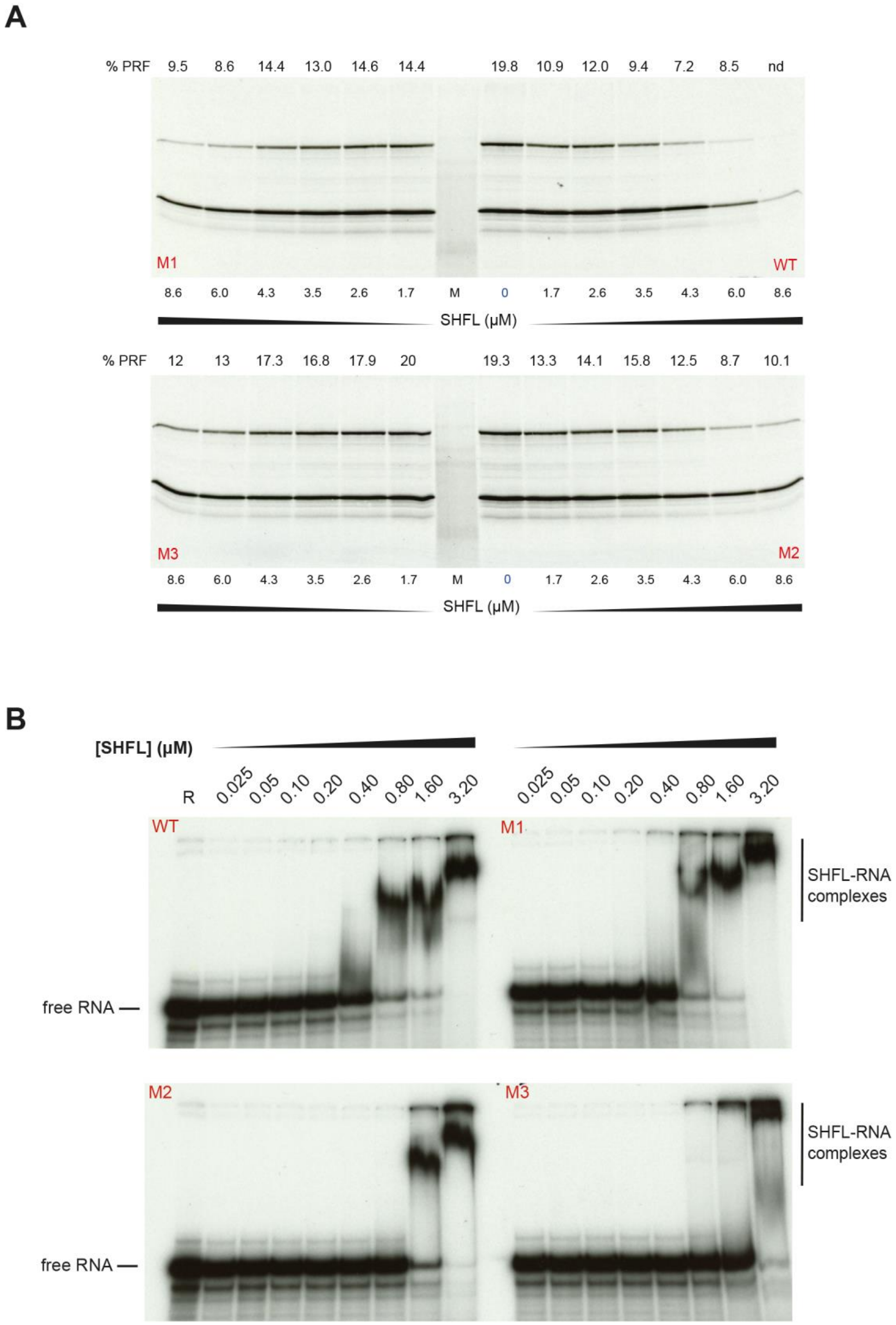

Figure S1: Original gels of Figure 6

Table S1: Intensities for stop and recoded species from Figure 3.

HIV-1

[SHFL] μM	*stop product (10)*	*PRF product (18)*	*% PRF*
0	84090	7355	4.5
1.7	81623	7338	4.6
2.6	76216	7021	4.7
3.4	77796	6422	4.2
5.2	71023	4578	3.4
6.9	32171	1486	2.5
8.6	14226	849	3.1

SARS-CoV-1

[SHFL] μM	*stop product (10)*	*PRF product (18)*	*% PRF*
0	67112	36823	19.7
1.7	91128	25846	12.3
2.6	75614	20952	12.1
3.4	88379	22919	11.4
5.2	83669	15454	8.7
6.9	94661	12804	6.6
8.6	68457	8091	5.7

IBV

[SHFL] μM	*stop product (10)*	*PRF product (19)*	*% PRF*
0	44514	30511	21.4
0.35	51022	26122	17.8
0.7	49601	23432	16.9
1.0	46998	22755	17.2
2.1	44298	15767	13.8
2.8	39711	13247	13.2
3.4	33470	10366	12.5
5.2	32886	9985	12.2
6.9	18763	5948	13.3

EMCV

[SHFL] μM	*stop product (9)*	*PRF product (19)*	*% PRF*
0	28378	24472	21.9
0.9	33793	19494	17.3
1.7	34213	10909	11.5
2.6	34019	8163	9.2
3.5	31800	5992	7.5
4.3	26266	5126	7.7
5.2	16916	3691	8.5
6.0	16310	4458	11.3
6.9	10213	1888	7.4
10.3	6659	1769	9.9

TMEV

[SHFL] μM	*stop product (10)*	*PRF product (19)*	*% PRF*
0	1176	10119	47.2
0.8	3958	7028	32.4
1.6	5013	4421	24.7
3.2	9984	3066	12.4
[SHFL M1] μM	*stop product (10)*	*PRF product (19)*	*% PRF*
0	2877	21842	46.5

0.8	3012	27856	47.5
1.6	3687	23122	45.4
3.2	3792	20154	44.3

MuLV

[SHFL] μM	*stop product (10)*	*RT product (14)*	*% RT*
0	14291	6738	22.8
0.35	11075	6339	26.0
0.7	13664	8652	27.7
1.0	13807	7323	24.8
1.4	15566	5153	17.8
2.1	13081	3512	15.1
2.8	14671	4177	15.8
3.4	15450	3433	13.0
5.2	10763	2045	11.4
6.9	9592	1317	8.6
8.6	8963	862	6.2

Phosphorimager values for the gels of Figure 3. Numbers in brackets indicate the number of methionines in the stop and recoded products.
